# Efficacy of *iSupport-Brasil* for unpaid caregivers of people living with dementia: protocol for a randomized and controlled clinical trial

**DOI:** 10.1590/1980-5764-DN-2023-0040

**Published:** 2023-11-24

**Authors:** Aline Cristina Martins Gratão, Anabel Machado Cardoso, Ana Carolina Ottaviani, Camila Rafael Ferreira Campos, Déborah Cristina de Oliveira, Diana Quirino Monteiro, Elizabeth Joan Barham, Fabiana de Souza Orlandi, Gustavo Carrijo Barbosa, Keila Cristianne Trindade da Cruz, Larissa Corrêa, Luana Aparecida da Rocha, Ludmyla Caroline de Souza Alves, Luiza Barros Maciel, Lucélia Silva Nico, Maria Cristina Corrêa Lopes Hoffmann, Sofia Cristina Iost Pavarini

**Affiliations:** 1 Universidade Federal de São Carlos, Departamento de Gerontologia, São Carlos SP, Brazil.; 2 Universidade Federal de São Carlos, Programa de Pós-Graduação em Enfermagem, São Carlos SP, Brazil.; 3 Universidade Federal de São Carlos, Programa de Pós-Graduação em Psicologia, São Carlos SP, Brazil.; 4 Universidad Andrés Bello, Faculty of Nursing, Campus Viña del Mar, Chile.; 5 Millenium Institute for Care Research, Santiago, Chile.; 6 Universidade Federal de São Carlos, Departamento de Psicologia, São Carlos SP, Brazil.; 7 Universidade de Brasília, Faculdade de Ciências Médicas, Departamento de Enfermagem, Brasília DF, Brazil.; 8 Ministério da Saúde, Coordenação de Saúde da Pessoa Idosa na Atenção Primária em Saúde, Brasília DF, Brazil.

**Keywords:** Caregivers, Internet-based intervention, Clinical Trial, Dementia, Cuidadores, Intervenção Baseada em Internet, Ensaio Clínico, Demência

## Abstract

Unpaid caregivers of people living with dementia tend to suffer mental health problems as a result of the negative effects associated with the care tasks. Thus, psychosocial interventions for this population group are necessary. iSupport is an online support program for caregivers that was created by the World Health Organization.

**Objective:**

To describe the design of a randomized clinical trial to measure the efficacy of the *iSupport*-*Brasil* version on caregivers’ mental health and well-being.

**Methods:**

The participants will be randomized into Intervention Group (IG) (n=195) and Control Group (CG) (n=195). For three months, the IG will access the *iSupport-Brasil* platform, the CG will enter the electronic page of the Brazilian Alzheimer’s Association, and both groups will be emailed the preliminary version of the “*Guia de cuidados para a pessoa idosa*” e-book (a guide to providing care to the elderly) from the Ministry of Health. The data will be collected at three moments: baseline, and three and six months after the beginning of the intervention.

**Results:**

It is expected that it will be possible to provide diverse validity evidence about *iSupport-Brasil* as an online and free intervention alternative, as a preventive means and as a way to promote mental health among caregivers of people living with dementia.

**Conclusion:**

Through the evaluation protocol of this randomized clinical trial on the effects of the *iSupport-Brasil* program, it may become a reference for countries that plan to adapt and improve the iSupport program using digital health solutions.

## RESUMO.

Cuidadores informais de pessoas que vivem com demência tendem a sofrer problemas de saúde mental como resultado dos efeitos negativos associados às tarefas de cuidado. Assim, intervenções psicossociais para essa população são necessárias. O iSupport é um programa de apoio *online* para cuidadores criado pela Organização Mundial da Saúde.

**Objetivo:**

Descrever o desenho de um ensaio clínico randomizado para medir a eficácia da versão iSupport-Brasil na saúde mental e no bem-estar de cuidadores.

**Métodos:**

Os participantes serão randomizados em Grupo Intervenção (GI) (n=195) e Grupo Controle (GC) (n=195). Durante três meses, o GI acessará a plataforma iSupport-Brasil, o CG entrará na página eletrônica da Associação Brasileira de Alzheimer e ambos os grupos receberão por *e-mail* a versão preliminar do *e-book Guia de cuidados para a pessoa idosa*, do Ministério da Saúde. Os dados serão coletados em três momentos: linha de base, três e seis meses após o início da intervenção.

**Resultados:**

Espera-se que seja possível fornecer diversas evidências sobre os efeitos do iSupport-Brasil como alternativa de intervenção *online* e gratuita, de forma a promover a saúde mental entre os cuidadores de pessoas que vivem com demência.

**Conclusão:**

O protocolo de avaliação deste ensaio clínico randomizado sobre os efeitos do programa iSupport-Brasil pode se tornar uma referência para os países que planejam adaptar e melhorar esta intervenção, usando soluções digitais de saúde.

## INTRODUCTION

Dementia is the seventh leading cause of death among all diseases and one of the main causes of disability and dependence among older adults, and generates the highest care-related costs in the last five years of life^1,2^. Unpaid caregivers of people living with dementia (PwD) are often exposed to risk factors regarding their physical and mental health, including high care demand, financial, social and leisure limitations and a constant need to manage the disease symptoms, among others^3,4^.

In this way, the importance of expanding well-being strategies for caregivers is highlighted^5^. Online interventions can be a good option; thus, in 2016, the World Health Organization (WHO) created the generic version of the iSupport for Dementia program, to be culturally adapted to its member countries^6,7^. iSupport for Dementia is an online psychoeducation program that aims at promoting emotional well-being for unpaid caregivers — family members or other unpaid people — of PwD in order to improve their quality of life^7,8^. In Brazil, the transcultural adaptation of the program, the evaluation of its usability, and its acceptability were already performed^9,10^. The next stage is to verify the effects of the program on caregivers’ mental health and well-being. The objective of this article is to describe the protocol of a randomized clinical trial (RCT) to be conducted in Brazil to verify the efficacy of the Brazilian version of the iSupport program on the mental health and well-being of caregivers of PwD.

## METHODS

### Study design

**Figure 1:**
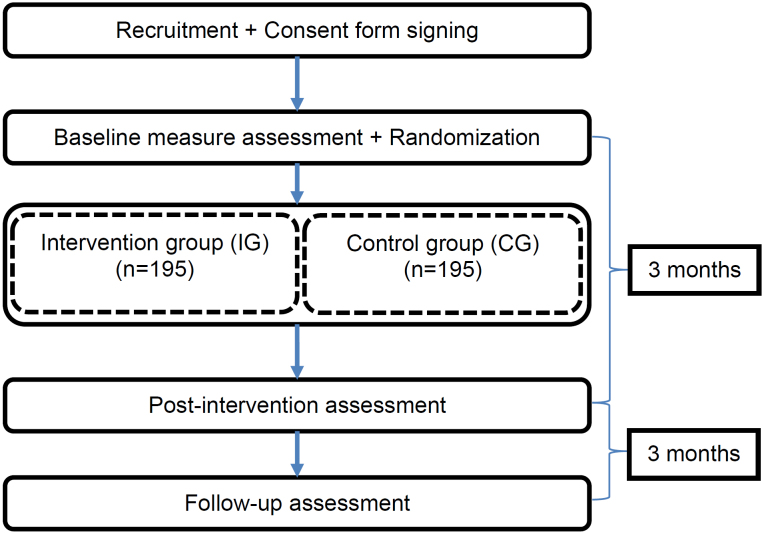
Flowchart corresponding to the stages of the randomized clinical trial to assess the effects of *iSupport-Brasil*.

A controlled, two-armed, and multicenter RCT will be conducted in Brazil (Figure 1), according to the guidelines set forth in the Consolidation Standards of Reporting Trials (CONSORT).

### Ethical considerations

The study was approved by the Human Research Ethics Committee of the Federal University of São Carlos (Opinion No. 5,141,963). All ethical procedures for research in human beings will be respected.

### Participants

#### Eligibility criteria

The subjects to be considered will be unpaid caregivers of PwD (including family members, friends, and neighbors), aged at least 18 years, living anywhere in Brazil, and providing care to a person diagnosed with dementia (self-report and Ascertaining Dementia Interview — AD8≥2) on an unpaid basis for at least six months at the time of recruitment. They should also have access to the Internet, as well as a score ≥4 on one of the three primary outcome measures: burden (single item), depression, or anxiety symptoms. Illiterate participants will be excluded, as well as those unable to understand written Brazilian Portuguese, and those who do not electronically accept the Free and Informed Consent Form (FICF).

#### Sample calculation

The calculation to determine the sample size was guided by the expected change in burden levels with Zarit Burden Inventory (ZBI) — six months after the baseline measurement. Alpha was set at 0.025 and the statistical power at 90%, and the minimum mean change index in ZBI was calculated based on findings from previous studies (mean change of 5.4). Thus, the minimum sample size for each group was calculated at 150 participants. Considering the mean sample loss rate in longitudinal studies^11^, this value was increased by 30% (n=390; 195 per group).

### Recruitment

Caregivers will be recruited through a continuous flow, through online and offline social communications, with the member associations of the Brazilian Alzheimer’s Association (ABRAz), and with public health services. First, the caregivers will electronically receive information leaflets about the study and a link to a digital platform to indicate their agreement with the FICF. Subsequently, they will be asked to answer a screening questionnaire to analyze the eligibility criteria. If they meet these criteria, the participants will answer the baseline form, along with other questions that will be used in the initial evaluation phase and will be informed via email about the randomization result. One of the researchers will direct the participants to use the link to the indicated website (depending on whether allocated to the IG or to the CG) and will help the caregivers to understand how to access all the available information.

### Randomization

Randomization will have a 1:1 allocation ratio between both groups and will be in charge of an independent researcher by means of a computerized random number generator. Allocation will take place after screening with eligible participants. The participants will be stratified based on the following characteristics: gender, age, and degree of kinship with the person cared for.

### Assessments

All participants will be assessed with the same set of instruments at the initial evaluation (screening + baseline), three months later (post-intervention measurement), and six months after the baseline evaluation (follow-up) (Table 1). The participants will receive email reminders in case they do not answer the questionnaire within a week.

**Table 1:** Overview of the measures for the randomized clinical trial corresponding to *iSupport-Brasil*.

Instrument		Result measures	No. of items	Initial assessment (T0)	Post-intervention (T1)	Follow-up (T2)
Screening						
	Demographic data	Gender, age, state of residence, marital status, schooling (years), ethnicity/race, family income, number of people in the household, paid activity.	9	X		
	Information about the care context	Degree of kinship, care time per week, caregiver hours per day, main caregiver, sharing the care task, regularly taking care of other people, living with the person cared for, and type of dementia diagnosed	8	X		
	HADS*	Depression and anxiety symptoms	14	X	X	X
	AD8	Screening for dementia	8	X	X	X
	Single item	Burden	1	X	X	X
Primary outcomes						
	ZBI	Burden	22	X	X	X
Secondary outcomes						
	QoL-AD	Quality of life	13	X	X	X
	RIS	Care self-efficacy	10	X	X	X
	PMS	Care mastery	7	X	X	X
	PBQ	Problematic behavior	3	X	X	X
	RMBPC	Problematic behavior	24	X	X	X
	PAC	Positive aspects	9	X	X	X
	DRS (caregiver)	Positive aspects	6	X	X	X
	SUS†	Usability	10	X	X	X

HADS: Hospital Anxiety and Depression Scale; AD8: Ascertaining Dementia Interview; ZBI: Zarit Burden Inventory; QoL-AD: Quality of Life - Alzheimer Disease; RIS: Relational, Instrumental and Self-care Scale; PMS: Pearlin Mastery Scale; PBQ: Problem Behavior Questionnaire; RMBPC: Revised Memory and Behavior Problems Checklist; PAC: Positive Aspects of Caregiving; DRS: Dyad Relationship Scale (caregiver version); SUS: System Usability Scale.

* HADS will be used both as a screening instrument and to assess the primary outcomes

† SUS will only be applied to the caregivers allocated to the Intervention Group.

#### Screening

The participants will be evaluated regarding sociodemographic data (date of birth, state in which they live, marital status, schooling, ethnicity/race, income, how many people they live with, whether they have any paid work), care (relationship with the person cared for, if they are the main caregiver, if they share care with someone else, if they take care of more people, how long they have provided care in years, days and hours, with whom the PwD lives, and the type of dementia), and technology use (equipment used to access the Internet and level of difficulty using it).

Dementia screening will assess whether the caregiver reports that the person under their care has cognitive impairments through the AD8-Brazil. This is a self-report questionnaire with eight items that is answered by the caregiver, who identifies functional decline attributable to cognitive impairment over the years. Its answer format is as follows: “Yes, there was a change”; “No, there was no change”; and “I don’t know”. The total score is the result of adding up the items with positive answers. A previous study showed excellent discriminant validity of 0.861 when compared to the scores obtained in the Clinical Dementia Rating Scale (CDR), 83.1% sensitivity, and 68.7% specificity^12^. Therefore, caregivers will be eligible for this study when they rate the person they care for with ≥2 points on this scale or mark any alternative other than the “I don’t know” option about the type of dementia that the person cared for has.

Burden will be assessed by means of the single-item Burden Scale, which evaluates the level of burden due to the care experience, where the caregivers can answer with values between 1 (no burden) and 10 (extreme burden). Depression and anxiety symptoms will be assessed by means of the Hospital Anxiety and Depression Scale (HADS). The instrument consists of 14 items, with seven targeted at assessing anxiety and the other seven for depression, using a Likert-type scale varying from 0 (zero) to 3 points. The total score varies from 0 to 21 points for each subscale, with higher scores indicating more severe symptoms^13–15^. For inclusion in the current study, the score should be ≥4 for burden (1 item) or depression or anxiety symptoms. Participants with severe burden, depression, and/or anxiety symptoms will be referred to mental health services for monitoring.

#### Primary outcomes

The primary outcomes will be measured by the symptoms of depression and anxiety with the HADS (described in the subtitle Screening) and the subjective burden with the ZBI. The ZBI version with 22 items measures the emotional, social, and financial burden of taking care of a person. Each of the items will be scored based on a 5-point Likert scale from 0 (never) to 4 (almost always). The total score varies from 0 (zero) to 88, with higher scores indicating a perception of more burden (Cronbach’s α=0.89)^16^.

#### Secondary outcomes

Quality of life will be assessed by means of the Quality of Life — Alzheimer Disease (QoL-AD) tool for people living with Alzheimer’s Disease (AD) and their respective caregivers/family members. The instrument consists of 14 items quantified in a 4-point scale, where score 1 is assigned to poor qualification and score 4, to excellent. The total score varies from 13 to 52, with higher values indicating better quality of life^17^.

The “care mastery” variable will be assessed with the Pearlin Mastery Scale (PMS), which verifies the extent to which the caregivers believe having care dexterity. It consists of seven items that are scored using a 4-point Likert scale, ranging from 1 (I totally disagree) to 4 (I totally agree), with a total score obtained by adding up all items and that can therefore vary from 7 to 28 points^18^. Scores closer to 28 indicate that the person feels in control of the care situations (internal mastery). Scores closer to 7 indicate that the person feels the situations are out of their control (external mastery)^19^.

To assess self-efficacy, the Relational, Instrumental and Self-care (RIS) Eldercare Self-efficacy scale will be used, which evaluates the caregivers’ perception about their ability to deal with the care tasks. Each of the ten items is classified with a 5-point Likert scale from 1 (I’m certain that I can’t do that) to 5 (I’m certain that I can do that). Higher scores indicate more self-efficacy^20^.

The Revised Memory and Behavior Problems Checklist (RMBPC) will be used to measure behavioral and memory problems related to dementia and the caregivers’ reactions to these situations. A total of 24 different conditions are presented, in which the caregiver must assess whether each problem is present or absent (with yes or no questions) and then assess their “reaction” or the extent to which they were “bothered” or “distressed” by every behavior found. The reactions are evaluated on a Likert scale from 0 (not at all) to 4 (extremely), so that the final score to assess the caregiver’s reactions can vary from 0 to 96 points^21^.

In addition to that, to identify PwD behaviors that the caregiver considers problematic and how to deal with them, the Problem Behavior Questionnaire (PBQ) will be used, adapted from Campos^22^. This is an instrument with three questions assessing the (a) problem behaviors related to dementia; (b) coping strategies used by the caregiver to deal with such situations; and (c) feelings perceived by the caregiver related to this role. The score is determined separately for each question. To analyze the results referring to questions *a* and *b*, the number of reported behaviors and strategies will be counted. For the *c* question, the score will be obtained from the difference between the number of positive and negative feelings reported by the caregiver^22^.

The positive aspects of care will be evaluated using the Positive Aspects of Caregiving (PAC) tool, which consists of nine items on a 5-point scale (from 1 to 5), scoring from 9 to 45 points, where higher scores represent more positive assessments^23^. The evaluation of aspects of the positive interaction in the caregiving experience will be measured by the Positive Interaction subscale, taken from the Dyad Relationship Scale (DRS-PI), caregiver version. The positive interaction factor consists of six assertions referring to the quality of the relationship between the dyad members and to the changes resulting from the care situation. It is a 4-point Likert scale (from 1=I totally disagree to 4=I totally agree). The final score is obtained from the mean score of all the questions in this factor, where the closer to four the higher the positive interaction degree^24,25^.

The System Usability Scale (SUS) will be used to assess usability of the *iSupport-Brasil* program for the experimental group only, at the end of the post-intervention, three months after the initial evaluation. SUS consists of ten items with a 5-point Likert scale (from 1=I totally disagree to 5=I totally agree). For the even questions, the score obtained is subtracted from five. For the odd questions, it is necessary to subtract one point from the score obtained. These “adjusted” values are added up and the final score is obtained by multiplying the sum by 2.5, with results varying from 0 to 100, where values lower than 68 indicate problems in usability of the system^26–29^. In addition, data such as the number of completed lessons, mean time to finish each lesson, and satisfaction with use of the program will be captured.

### Intervention

The participants in each group will have access to their respective material for a period of three months from the moment they receive the link. All of them will be instructed to use the indicated material at least once a week and for at least one hour. At any moment of the intervention, they will be able to contact (via email) the research team to solve eventual doubts. The entire recruitment, data collection, and intervention process will be conducted online.

#### Intervention group

**Figure 2:**
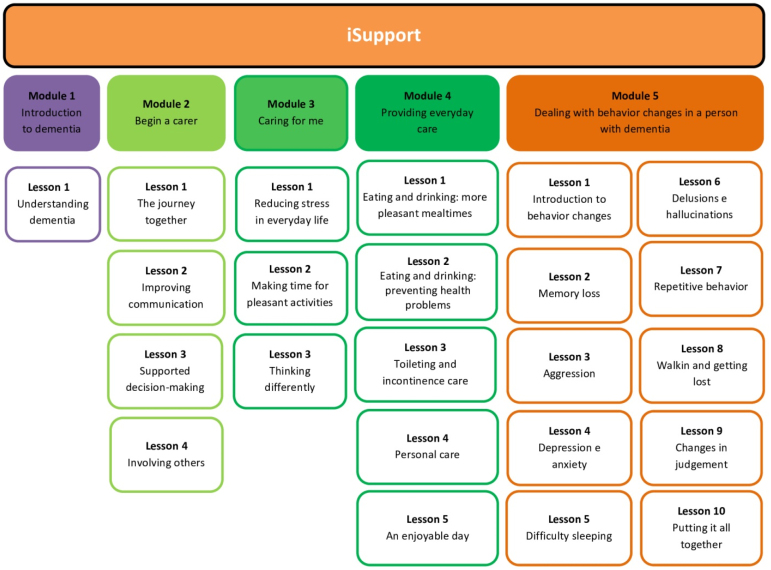
Overview of the *iSupport* self-help training and support online program: structure and content.

The intervention will involve access to the *iSupport-Brasil* platform, an online psychoeducation support program for caregivers that use interactive technological resources. For example, a given care-related problem is described and the caregivers must choose between different alternatives which one they understand is the best behavior in the face of a specific situation. When the alternative selected is not ideal to solve the problem, the caregivers immediately receive information explaining why the option is not the most suitable for greater success and better self-efficacy. Additionally, some activities are proposed for caregivers to observe and analyze the interactions that occur with their family member who lives with dementia and decide what they can do to reduce some of the difficulties they face. The program is also personalized, in the sense that, besides the mandatory topics, the caregivers choose the ones of greater interest. In addition to that, the names of the caregivers and the persons under their care, recorded when they sign up for iSupport, are incorporated into the examples used in the program, personalizing the interaction, and increasing the relevance and impact of the activities for the participants. The use of each module will be registered on the central *iSupport-Brasil* platform by the Federal University of São Carlos (Universidade Federal de São Carlos — UFSCar) and currently under the control of the Ministry of Health. The program has five modules, divided into 23 lessons as shown in Figure 2.

#### Control group

The CG activity will involve using the existing and publicly accessible ABRAz educational website (https://abraz.org.br) to disseminate knowledge about AD, and other dementias, and offer social, emotional, and informational support to family members, among other topics. The ABRAz website is focused on information and not on skills training. There will be no restriction on which other websites the CG participants will be able to access. Also, the GC participants will be emailed the preliminary version of the “*Guia de Cuidados para a Pessoa Idosa*” e-book from the Ministry of Health^30^. This e-book is divided into four modules with guidelines and basic information on the care required by aged individuals, for self-care, family caregivers, and interested parties.

#### Statistical analysis

For the efficacy analysis, initially, the data distribution normality will be verified for all instruments. Variables that present values >0.05 in the Kolmogorov-Smirnov test will be considered parametric tests^31^. Subsequently, based on multiple analyses of variance (with repeated measures), comparisons will be made for dependent samples (within groups: MANOVA or Friedman) and for independent samples (between groups: ANOVA or Kruskal-Wallis) for the data obtained in each of the three data collection waves (initial assessment, post-intervention measurement, and follow-up). A 95% confidence interval will be considered, with alpha set at <0.05^31^. The effect size of the changes observed will be obtained from Cohen’s formula, as follows:

- small: values below 0.20;
- average: values between 0.2 and 0.8; and
- large: values above 0.80^32^.

For qualitative analyses, two judges will agree on the choice of categories and subsequent data categorization^33^. The Internet data will be analyzed using each metric (e.g., use frequency of parts of the *iSupport-Brasil* platform through Google Analytics).

## RESULTS

The strategies described in this study are expected to be effective for recruiting and assessing the effects of *iSupport-Brasil* on the mental health and well-being of Brazilian unpaid caregivers of PwD. It is expected that *iSupport-Brasil* exerts positive effects, statistically significant and with good effect size in the IG when compared to the CG, both in the post-intervention period and in the maintenance of gains (follow-up).

## DISCUSSION

This protocol describes the stages of an RCT from a WHO program for unpaid caregivers of PwD, translated and adapted for the Brazilian context: *iSupport-Brasil* ^34^. Designing and evaluating programs with this objective is a public policy priority, as caregivers of PwD tend to present worse mental health conditions than non-caregivers^35^. Supporting caregivers of PwD is one of the priority areas of the WHO Global Action Plan for a public health response to dementia (2017–2025), to which Brazil is a signatory^36^. Consistent with this proposal, *iSupport-Brasil* should be included in the Research Priorities Agenda in Brazil^37^. In addition to being an increasingly common function among people due to the exponential increase in cases of dementia, currently, unpaid caregivers frequently report it as a task that imposes burden^38^. Furthermore, there are few psychoeducation programs designed for caregivers with proven efficacy, which implies the need to develop rigorous protocols for evaluating these interventions^39^.

It is believed that *iSupport-Brasil* will help minimize the support demand for unpaid caregivers of PwD in Brazil, as it is an innovative program for this population segment. Offering an intervention that presents scientific evidence is essential to guarantee its quality and effectiveness. In this sense, when measuring the effects of *iSupport-Brasil* on the caregivers’ mental health and well-being, it will be possible to outline in which aspects this program tends to contribute to the life of the caregivers and the people cared for, as well as to know its limitations and reflect on possible alternatives.

As for social importance, at the end of this study, the *iSupport-Brasil* program will be made available free of charge for all caregivers who seek better mental health conditions and wish to gain knowledge about dementia and improve their skills on how to care for people living with this disease.
